# Computational Design of Hypothetical New Peptides Based on a Cyclotide Scaffold as HIV gp120 Inhibitor

**DOI:** 10.1371/journal.pone.0139562

**Published:** 2015-10-30

**Authors:** Apiwat Sangphukieo, Wanapinun Nawae, Teeraphan Laomettachit, Umaporn Supasitthimethee, Marasri Ruengjitchatchawalya

**Affiliations:** 1 Bioinformatics and Systems Biology program, King Mongkut’s University of Technology Thonburi (KMUTT), Bang Khun Thian, Bangkok, 10150, Thailand; 2 Pilot Plant Development and Training Institute, KMUTT (Bang Khun Thian), Bangkok, 10150, Thailand; 3 Biotechnology program, School of Bioresources and Technology, KMUTT (Bang Khun Thian), Bangkok, 10150, Thailand; 4 School of Information Technology, KMUTT, Bang Mod, Thung Khru, Bangkok 10140, Thailand; Wake Forest University, UNITED STATES

## Abstract

Cyclotides are a family of triple disulfide cyclic peptides with exceptional resistance to thermal/chemical denaturation and enzymatic degradation. Several cyclotides have been shown to possess anti-HIV activity, including kalata B1 (KB1). However, the use of cyclotides as anti-HIV therapies remains limited due to the high toxicity in normal cells. Therefore, grafting anti-HIV epitopes onto a cyclotide might be a promising approach for reducing toxicity and simultaneously improving anti-HIV activity. Viral envelope glycoprotein gp120 is required for entry of HIV into CD4+ T cells. However, due to a high degree of variability and physical shielding, the design of drugs targeting gp120 remains challenging. We created a computational protocol in which molecular modeling techniques were combined with a genetic algorithm (GA) to automate the design of new cyclotides with improved binding to HIV gp120. We found that the group of modified cyclotides has better binding scores (23.1%) compared to the KB1. By using molecular dynamic (MD) simulation as a post filter for the final candidates, we identified two novel cyclotides, GA763 and GA190, which exhibited better interaction energies (36.6% and 22.8%, respectively) when binding to gp120 compared to KB1. This computational design represents an alternative tool for modifying peptides, including cyclotides and other stable peptides, as therapeutic agents before the synthesis process.

## Introduction

Cyclotides, which represent a large group of triple disulfide macrocyclic peptides [1–3], possess 28–37 amino acid residues that can be divided by successive Cys residues to form six consecutive loops. The triple cystine knot structure, which is conserved among the cyclotide family, provides remarkable stability against extreme thermal and chemical conditions as well as enzymatic degradation [4]. In addition, a broad range of biological activities, including uterotonic, insecticidal, cytotoxic, and anti-HIV activities have been described [5]. Accordingly, the cyclotide molecules are an attractive platform for drug design applications. For example, engineered cyclotides can have high oral bioavailability that is comparable to small molecule drugs while retaining desired target specificity of the grafted epitopes [6–9]. Moreover, cyclotides can be synthesized through chemical reactions [10] and genetic recombination in bacteria [11], which allows for a high yield of material.

Treating HIV remains one of the biggest challenges we face today. Several cyclotides have shown anti-HIV activity [12, 13], including KB1 [14]. However, the use of this class of compound as an anti-HIV therapy is limited due to high toxicity in normal cells [15]. The toxicity of KB1 was shown to involve residues in loops 5 and 6 [6, 16]. Therefore, grafting anti-HIV epitopes in between these loops might be a promising approach for reducing toxicity and simultaneously improving anti-HIV activity.

The gp120 is an HIV envelope glycoprotein that is required for attachment to the CD4 receptor present on human CD4 T cells and initiation of the HIV life cycle [17]. Although the three dimensional (3D) structure of gp120 has been available since 1998 [18], the development of drugs targeting gp120 remains challenging due to its high degree of variability and physical shielding [19]. Nevertheless, the gp120 surface possesses unique CD4 binding regions that could serve as potential therapeutic targets, since they are conserved among various strains of HIV and need to be exposed at least transiently for viral entry into the target cell [20].

An *in silico* method has been successfully used to design *de novo* peptides to target gp41, another glycoprotein of HIV [21]. In addition, functional motif grafting methods have been proposed to increase the experimental success rate of target-specific peptide production [22]. The challenge of protein grafting lies in the process of inserting biologically active epitopes onto an appropriate position in order to achieve the desired effects. In general, the method involves the following three common steps: identifying active epitopes, grafting the epitopes onto the scaffold, and validating the activity of the grafted scaffold [22, 23]. In the first step, the functional motifs that display strong interaction with the target protein were identified. The motif usually contains hotspot regions, which are amino acid residues that largely contribute to a pair of protein-protein interaction [24–26]. The hotspot residues are then integrated into a scaffold, which is normally a stable peptide such as KB1. To maintain the original activity, the hotspot motifs must be grafted into a suitable position on the scaffold. However, identifying suitable positions is not easy and requires exhaustive search methods.

In contrast, genetic algorithm (GA) is a heuristic search method based on Darwin’s theory of evolution [27]. GA uses four unique components together: parallelism, selection, mutation, and crossover to deliver solutions. The major advantage of GA is the ability to conduct a parallel search, which can explore multiple possible solutions in the solution space at the same time to avoid local sub-optima [28]. GA has been applied to a wide range of problems, including automated drug design, but mostly with regard to small molecule modifications [29–31]. In the case of a peptide molecule, the combination of GA with molecular docking software has been used to automate *de novo* peptide design to target specific proteins [32–34]. These methods create new peptides containing 4–6 residues. Importantly, it has been postulated that the success of this approach depends on the quality of the docking mechanism used.

In this study, we attempted to reduce the undesirable effects and improve the anti-HIV activity of KB1. Loops 5 and 6 were selected as the targets for computational modification based on their important roles in membrane binding, membrane disruption [16, 35], and hemolytic activity [6]. We demonstrated that our automated design could generate potential cyclotides that interfere in the first step of the HIV entry cell process—gp120-CD4 interaction. Firstly, an automated cyclotide modification process based on GA and a molecular modeling pipeline was built. This involved the use of GA to select peptides based on CD4-gp120 hotspot regions for grafting onto KB1. In the evaluation method of GA, we created a computational pipeline that constructed the 3D structure of a modified KB1 using homology modeling, and then measured the binding affinity to gp120 by protein-protein docking prediction. We then performed high-resolution screening by using MD simulation to deeply evaluate the affinity of the modified cyclotide candidates for gp120 based on the GA step. The modified cyclotides were then assessed for their potential to interact with gp120 by comparing the interaction energy of gp120 and the cyclotides with gp120 and CD4-mimetic miniprotein (CD4M33), a gp120 inhibitor [36, 37]. Finally, we describe two modified cyclotides that show high potential for gp120 inhibition.

## Results and Discussion

### Cyclotide modification using GA and a molecular modeling pipeline

**Fig 1:**
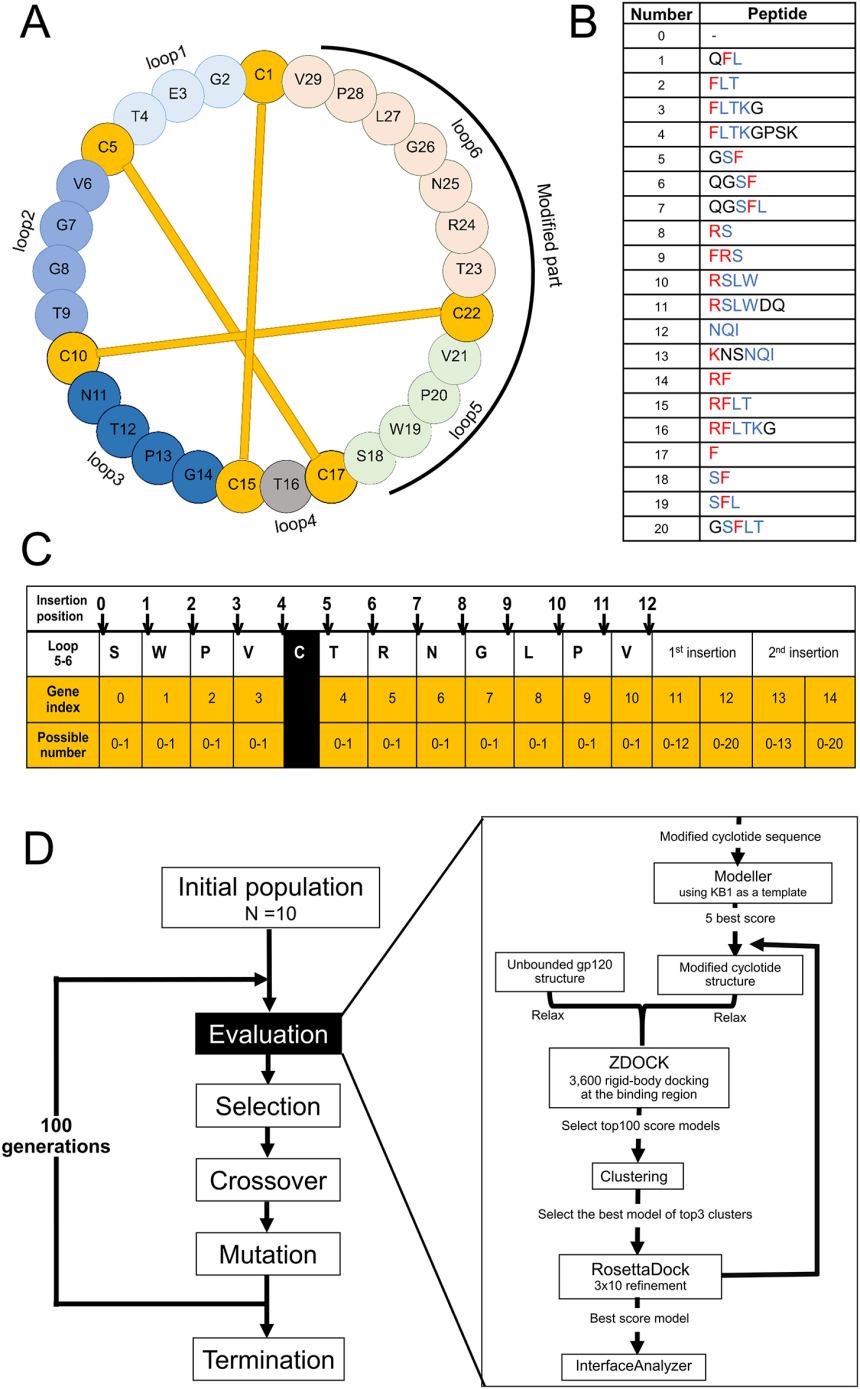
Genetic algorithm schema for modifying the KB1 sequence. (A) Native KB1 primary sequence. The disulfide bonds are linked by yellow lines. (B) Hotspot-containing peptide library. Residues in red show the contribution to binding energy lower than -2.0 kcal/mol; blue, binding energy in the range of -0.5 to -2.0 kcal/mol; black, binding energy higher than -0.5 kcal/mol (estimated by Anchor). (C) Chromosome design and configuration. Chromosome emphasized by yellow represents the operation on loop 5 and loop 6 of KB1. (D) Molecular pipeline of the GA process and evaluation. After the initial chromosomes are generated, the evaluation method (right box), which contains the molecular modelling pipeline, is implemented. The GA is processed with 100 generations and 10 individuals per generation.

Before grafting, hotspot residues of the CD4-gp120 interaction were identified as possible key residues of the new cyclotides for binding to gp120. Significant residues that contributed large binding energy (< -2.0 kcal/mol [24, 25] or -8.4 kJ/mol) as predicted by Anchor were Phe43, Arg59, and Lys29. This result corresponded with the prediction from HotRegion, which identified Phe43 and its neighbors (Gln40 and Leu44) as the cluster of the hotspot region. In agreement with these findings, Phe43 and Arg59 of CD4 were previously reported to be the most important residues involved in the gp120-CD4 interaction [18, 38–40]. The hotspot residues were identified as key residues that contribute to protein-protein interaction. However, some residues around the hotspot may facilitate the hotspot-target interaction [41]. Therefore, the residues located near the hotspot were also included in grafting. In the library, we generated the epitopes by including the hotspot region and its neighbor residues where the length of the epitopes was in a range 1–10 residues. Similar epitope design procedure has been used before in another study [6]. The hotspot-containing peptide library is provided in Fig 1 and S1 Table.

In the GA protocol, the peptides in the library were randomly selected to graft onto cyclotide KB1 scaffold as described in the Methods and Fig 1. The automated process was conducted by GA optimization, which attempts to minimize the binding score of the modified cyclotide and gp120. After 100 generations of GA, the interaction score (Rosetta binding score) generated from the binding of the modified cyclotides to gp120 was compared to the score of the native KB1-gp120 interaction (Table 1). The average binding score of the modified group (-5.01) was significantly lower approximately 23.1% (*i*.*e*., having higher affinity) than that of the native group (-4.07), suggesting that our GA protocol was able to create new cyclotides that had greater specificity to gp120 than the native structure. The generated group of cyclotides with scores better than those of the native structure (hereafter called “candidates”) consisted of 341 individuals, which represents 37% of the total cyclotides from the GA.

**Table 1:** The Rosetta docking score to gp120 of the modified cyclotides versus KB1.

Groups	Number of compared structure	Binding score (Rosetta unit)		
		Average	Maximum	Minimum
KB1^a^	8^b^	-4.07 (±0.76)	-3.08	-5.23
Modified KB1	910	-5.01 (±0.79)	-2.24	-7.95

^a^ global docking mode;

^b^ different orientations after clustering at the CD4-binding site

We next compared solvent accessible surface area buried at the interface (ΔSASA) and the proportion of the loop contribution at the interface (Table 2). We found that the buried area of the candidates that bound to gp120 was approximately 17% larger than the area of the native structure. This result suggests that the stronger interaction was due to the hotspot and its surrounding residues [24, 42] In addition, we found that the fraction of loop 5–6 residues present in the interaction interface (determined by InterfaceResidue script) of the modified group increased by approximately 18% and, accordingly, the fraction of loop 1–4 residues present at the interface decreased by approximately 13% compared to the native group. The increase of ΔSASA as well as the contribution of loops 5–6 indicate that our engineering process at this region on KB1 can enhance the binding activity to gp120.

**Table 2:** Binding interface properties in complex with gp120 of modified cyclotides versus KB1.

Groups	Average ΔSASA (Å^2^)	Average fraction of residues in the interaction interface (%)	
		Loop 1–4	Loop 5–6
8 orientations of KB1	1146.8 (±115.1)	57.0 (±14.1)	47.1 (±17.0)
341 modified cyclotides	1338.2 (±183.1)	43.7 (±22.6)	64.8 (±15.1)

### High-resolution screening of the candidates by MD simulation

Due to the limitation of rigid-body docking software and high flexibility of peptides [43], we attempted to improve the efficiency of the prediction by using an MD simulation as a post filter. The 10 best candidate structures ranked by Rosetta score were selected for further analysis. Of these, four cyclotides did not have three complete disulfide bonds (*i*.*e*., atomic distance of the sulfur between two cysteine residues was greater than 2.50 Å). The incomplete disulfide bond was a result of the homology modelling process, which constructs a new 3D model based on amino acid sequence alignment. Actually, the disulfide bonds were initially constrained by the template structure. However, the constrained structure might cause atomic clash and conflict. Thus, we further optimized the whole structures and refined protein loops using the Modeller software to yield more accurate structures. The process, however, may cause loss of disulfide bonds. After ranking the binding scores of all 910 structures, we found that the top 10 structures (See S2 Table) contain 4 structures (including the first-ranked structure) without complete disulfide bonds. This suggests that chromosomes whose structures lack in complete disulfide bonds, could bear genes with profitable affinity traits. In regard to the strategy of the GA, it is highly possible that these traits would be passed on to next-generation chromosomes whose structures have complete cyclotide bonds as a result of genetic operations such as mutation and crossover. Filtering out chromosomes with incomplete disulfide structures during the GA process would lead to the search effort that is prone to be suboptimal.

To investigate the affinity of the binding, the six modified cyclotides containing three complete disulfide bonds and the native KB1 (the best docking score) in complex with gp120 were simulated in water under neutral conditions for 20 ns. The distance between the center of mass indicated that the modified cyclotides as well as the native structure maintained gp120 binding (see S1 Fig). The affinity of the binding was determined by measuring the non-covalent interaction energy, which is a combination of electrostatic and VDW interaction energies. We observed that the energies of two modified cyclotides, GA763 (-1067 kJ/mol) and GA190 (-959 kJ/mol), were significantly lower than the native structure (-781 kJ/mol) (two sample Z-test; P < 0.0001) (Table 3). These results indicated that the modified cyclotides display better binding affinity than the native structure. GA763 was the best candidate with the lowest total energy, which is approximately 36.6% lower than that of the native structure. GA190 had a total energy which is approximately 22.8% lower than that of the native structure.

**Table 3:** Interaction energy and number of H-bond of modified cyclotides, KB1, and CD4M33 in binding gp120.

Inhibitors	H-bond	Average non-bonded interaction energy (kJ/mol)		
		Electrostatic energy	VDW energy	Total energy
GA763	3.7	-718 (±244)	-350 (±24)	-1067^a^,^b^ (±237)
GA190	9.9	-584 (±82)	-375 (±30)	-959 ^a^, ^b^ (±91)
GA689	4.1	-326 (±122)	-247 (±21)	-574 (±125)
GA479	6.4	-275 (±56)	-258 (±20)	-532 (±59)
GA218	5.2	-355 (±178)	-167 (±27)	-522 (±184)
GA61	2.7	-187 (±52)	-226 (±18)	-413 (±53)
KB1	6.1	-448 (±66)	-333 (±22)	-781 (±68)
CD4M33	5.3	-538 (±143)	-299 (±25)	-836^a^ (±149)

The interaction energy was calculated from the sum of average electrostatic and Van Der Waal (VDW) energy in the last 5 ns in a 20 ns MD simulation. The number of H-bond was the average number of H-bond per time frame in the last 5 ns.

^a^ Significantly different from the native by two sample Z-test;

^b^ Significantly different from CD4M33 by two sample Z-test

We also compared the binding energies of these two cyclotides to that of the 27 amino acid CD4 mimic, CD4M33 [36, 37]. Interestingly, the non-bonded interaction energy of CD4M33 binding to gp120 was -836 kJ/mol, which was significantly higher than those of the two cyclotides (Table 3). These results indicated that the binding energies of the engineered cyclotides improved over that of the native structure and were comparable to the known inhibitor. We next compared the backbone root-mean square deviation (RMSD) and root-mean square fluctuation (RMSF) (see S2 and S3 Figs, respectively) of the native and modified groups and found that the modified cyclotides exhibited more fluctuation than native KB1, particularly in loop 6. Therefore, this limitation of the candidates should be improved, such as by applying a flexible docking method instead. However, time efficiency is one advantage of using the rigid-backbone docking method.

### Two potential candidates for gp120 inhibition

**Fig 2:**
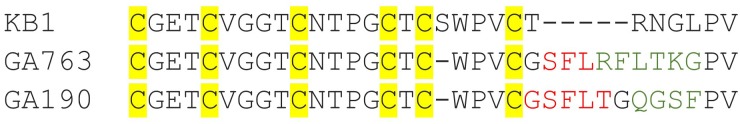
The sequence alignment of KB1, GA763 and GA190. The first and second hotspot-containing peptides are represented in red and green, respectively. Cysteine residues are highlighted by yellow.

The amino acid sequences of GA763 and GA190 were different from the native, especially in loop 6 (Fig 2). The pattern of the deletion in both modified cyclotides was the same, whereby Ser18, Thr23, Arg24, Asn25, and Leu27 were deleted. The hotspot-containing peptides were inserted only into loop 6: “SFL” and “RFLTKG” were grafted into GA763, and “GSFLT” and “QGSF” were grafted into GA190. In order to identify the residues important for binding, we next investigated the interaction energy of each residue on the two modified cyclotides.

### GA763

**Fig 3:**
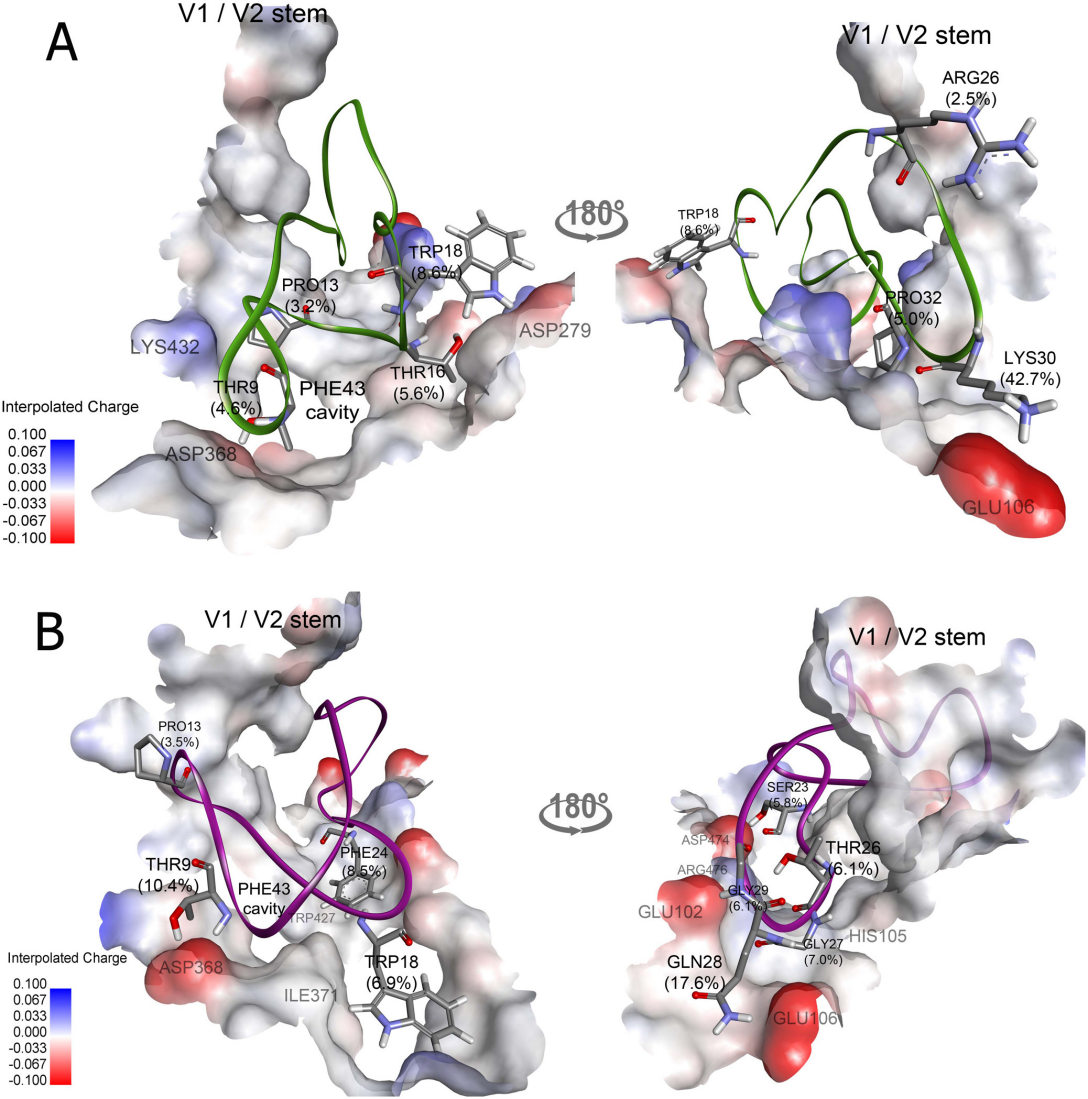
The binding of two candidate cyclotides with gp120. (A) GA763 and (B) GA190 in gp120 binding pocket are presented in green and purple ribbons, respectively. The important residues for binding to gp120 are shown in stick with the residue name and percent contribution of the non-bonded interaction energy. The gp120 is represented by an interpolated charge surface. Structures are rendered by Discovery Studio Visualizer.

Surprisingly, almost half (42.7%) of the interaction energy of GA763 was due to Lys30 (Fig 3A), which is an amino acid in the inserted peptide. This lysine resided near Glu106 of gp120 and resulted in large electrostatic interaction as well as possible salt bridge formation. The other residues, including Thr9, Thr16, Trp18, and Arg26, also participated in electrostatic interactions. In particular, Trp18 contributed to 8.6% of the total energy. Although Trp18 is hydrophobic, its indole nitrogen was able to donate hydrogen to Asp279, resulting in high electrostatic interaction and hydrogen bond formation. The VDW interaction energy of Pro13 was lowest among all residues (3.2% of the total energy), which might be the result of a hydrophobic effect around the Phe43 cavity (the conserved-hydrophobic region of gp120) [18]. The average number of hydrogen bonds per timeframe of GA763 was approximately three, which was rather sparse compared to the others. When the number of hydrogen bonds was decomposed, Trp18 provided the greatest contribution (approximately 1.3 bonds on average per time frame). The interaction energy of each residue of GA763 is shown in S3 Table.

### GA190

In contrast to GA763, the binding between GA190 and gp120 was due to several residues based on the distribution of interaction energy (Fig 3B). Gln28 (grafted within the ‘QGSF’ motif) had the greatest contribution in the interaction energy (17.6%), which was dominated by electrostatic interactions. This result was likely due to the location of Gln28 near the charged residues of gp120 (*i*.*e*., Glu102 and Glu106), which resulted in the formation of two hydrogen bonds on average. In addition, Thr9 in loop 2 electrostatically interacted with Asp368, resulting in the formation of approximately two hydrogen bonds. Moreover, we found that Phe24, which was inserted in the “GSFLT” motif, strongly interacted with the hydrophobic residues of the Phe43 cavity (*e*.*g*., Trp427). As a result, Phe24 was dominated by VDW energy and contributed approximately 8.5% of the total energy. In addition, Trp18 contributed a large proportion to the total energy (6.9%), which was similar to GA763, but the interaction was different. Trp18 exhibited high VDW forces that might have been due to the interaction with Ile371 and Val372. Interestingly, GA190 formed approximately 10 hydrogen bonds per time frame, which suggests that this binding has remarkable specificity [44, 45]. Based on the distribution of energy and the number of hydrogen bonds, GA190 likely interacts with gp120 through several residues, which is different from that observed with GA763. The interaction energy of each residue of GA190 is shown in S4 Table.

Comparing energy between loops, we found that loops 5 and 6 of both the modified cyclotides had larger contributed (~40%) to the interaction energy than that of loops 1–4 (Table 4). Also, the energy contribution of loop 5–6 of both cyclotides is increased ~6% compared to that of the native. Corresponding with Tables 1 and 2, the results support that the improvement of the gp120 interaction of the modified cyclotides is generated by using our GA process.

**Table 4:** Non-bonded energy contribution of loops 1–4 and loops 5–6 in cyclotides binding to CD4.

Cyclotides	Energy contribution (%)	
	Loop 1–4	Loop 5–6
KB1	36.3	63.8
GA763	30.2	69.8
GA190	29.7	70.3

## Conclusions

In this study, we generated new HIV gp120 peptide inhibitors based on a cyclotide backbone using automated modification with GA and a molecular modeling pipeline. The process used a combination of molecular modeling techniques to predict sequences and structures of novel cyclotides that can bind to the target, gp120. The hotspot-containing peptides were identified from CD4 interface residues and used in the interaction domain of the modified cyclotide. GA was applied to modify the sequences of cyclotides and to determine the optimal location for peptide grafting, and the fitness score was calculated from the pipeline programs Modeller, ZDOCK, and RosettaDock. The candidates were selected and validated by MD simulation. Finally, we described two novel cyclotides as potential HIV inhibitors whose non-bonded interaction energy was lower than that of both native KB1 and CD4M33, a known gp120 inhibitor. Our GA763 showed the lowest energy, which was approximately 37% lower than that of the native structure. The interaction was largely due to the inserted peptide, especially Lys30, which contributed almost half of the total energy. The other candidate, GA190, also had a binding energy that was approximately 22% lower than that of the native structure. One important feature of GA190 was the high number of hydrogen bonds (10 bonds per time frame), indicating the high binding specificity to the target. This computational design represents an alternative tool for modifying peptides, including cyclotides and other stable peptides, as therapeutic agents before the synthesis process.

## Methods

### Automated cyclotide modification using GA and a molecular modeling pipeline

We first determined hotspot residues of the gp120-CD4 interaction (Fig 1B) before grafting onto KB1 (Fig 1C). For grafting, automated protocol was then created by GA and evaluated for their affinity to gp120 using a molecular modeling pipeline (Fig 1D). The GA protocol was configured according to the framework of the distributed evolutionary algorithms in python (DEAP) version 1.0.0 [46]. The data were processed on an Intel i7-4770 desktop with 3.40 GHz and 16 GB RAM.

### CD4-gp120 hotspot determination

The hotspots of interaction between gp120 and its native partner, CD4, were determined using three different approaches, including HotRegion [47], ANCHOR [48], and a literature search. The HIV-1 gp120-CD4 complex (PDB ID: 1GC1) [18] was submitted as input into the online tools HotRegion and ANCHOR and hotspots were determined using the default parameter of each tool.

### Chromosome design and configuration

To implement GA, the trial solution was represented as a string or “chromosome”. In our case, the chromosome was designed to represent a modification of the KB1 sequence on loops 5 and 6. Our chromosome contained 15 segments or “genes”, which were categorized into three main operations: deletion, insertion, and peptide selection (Fig 1C). Gene indices 0 to 10 corresponded to positions of amino acid residues on loops 5 and 6. These genes stored the deletion operation, whereby if the trait value is 0, then the residue at the corresponding position will be deleted. In contrast, a value of 1 means that such residue will be retained. The position for peptide insertion was separated into 13 possible positions (0–12). Gene index 11 defines the position on the KB1 sequence for inserting the peptide from the peptide library, which is indicated by gene index 12. Since the hotspot residues of CD4 were not continuous, we also doubled the insertion command (gene indices 13 and 14). Note that the possible position for the second insertion is in the range of 0–13 because the position was updated from the first insertion. The chromosome generation and translation of the modified cyclotide sequence are described by pseudo code in S1 Text.

### Molecular pipeline for binding affinity evaluation

The quality of the modified cyclotides was evaluated by fitness score calculation based on their binding affinity to gp120 using our molecular modeling pipeline (Fig 1D). First, 3D structures of the modified cyclotides were constructed based on KB1 (PDB ID: 1NB1) [1] as a template by using the Modeller program (version 9.13) [49]. The entire structure was optimized with 10 repeated cycles of slow mode. Loop refinement was then applied (only for loops 5 and 6) with 50 model predictions in fast mode. After modeling, the five best structures ranked by discrete optimized protein energy (DOPE) scores were selected. Molecular docking was then performed between the generated structures and gp120.

Before the docking process, the structures were relaxed to reduce atomic crash using the fast relax application in Rosetta software (version 3.5) with the fixing backbone mode. Since the binding pocket of gp120 is conserved and need to be exposed to bind with CD4, the modified cyclotides were docked at only this region to reduce search space. The binding pocket area was determined by InterfaceResidues script (available at www.pymolwiki.org/index.php/InterfaceResidues), and the additional ZDOCK script (block.pl, available in the ZDOCK package, http://zdock.umassmed.edu/software) was applied to restrict the docking site. All hydrogen atoms were then removed from both gp120 and the modified cyclotides. The optimized cyclotide structures were then docked to gp120 at the CD4 binding site by using ZDOCK (version 3.0) [50]. ZDOCK was processed with 3,600 structure predictions and 15 degree sampling. The predicted structures were then ranked by their ZDOCK scores, and only the top 100 structures were selected for clustering. The Rosetta cluster application was applied by using a root-mean-square (RMS) cluster radius of 3.0 Å. All clusters were ranked by the Rosetta score and the cluster size, and the best scores from the top three clusters were selected for further refinement.

To yield more reliable structures, the initial docking state from ZDOCK were refined using RosettaDock [51], which is able to simultaneously optimize both rigid-body orientation and side-chain conformation [52]. The RosettaDock was used to predict 10 structures for each input ZDOCK structure. The parameters in the RosettaDock refinement protocol were set as default. The best score structure was selected to calculate the interaction score and ΔSASA with the InterfaceAnalyzer application in Rosetta. This interaction score was used as a fitness score in GA.

In the GA process, 10 chromosomes were generated for each generation. After the evaluation, the best chromosome was kept for the next generation. A tournament selection with tournament size of 3 was used as a selection method. The probability of crossover was set to 0.5. The probability of mutation of the chromosomes was set to 0.2 per gene, except that one random chromosome was set to mutate with a high probability (0.8 per gene) in order to improve the variety. GA was processed for 100 generations.

### gp120 and native cyclotide KB1 docking

The affinity between the native KB1 and gp120 at the CD4 binding site was determined by Rosetta global docking. Cyclotide KB1 and gp120 structures were downloaded from protein data bank with PDB ID of 1NB1 and 1GC1, respectively. The gp120 structure was separated from its complex. Both gp120 and KB1 structures were relaxed using the Rosetta relax application. RosettaDock was applied by using a global docking mode with 100,000 structure prediction and default parameter settings. After docking, the top 1000 structures were clustered with the Rosetta cluster application using a RMS cut-off of 3.0 Å. To select the native KB1 orientations at the CD4 binding site, the structures that contributed more than 50% of residues as the interaction interface at the binding region were selected. The InterfaceResidue script was used to investigate the interface residues at the binding region. The selected structures were then analyzed by Rosetta InterfaceAnalyzer.

### MD simulation of cyclotides

After the GA process, the top 10 structures ranked by the Rosetta score were chosen for further investigation on the stability, affinity, and key residues involved in binding using MD simulation. Before the simulations, structures that had lost the disulfide bond were filtered out. The disulfide bonds were detected by measuring atomic distance between sulfur with a cut off range of 2.5 Å. This criterion was set to avoid over fitting of the general definition of the disulfide bond length of approximately 2.05Å [53].

The MD simulations were performed using Gromacs software (version 4.6.7) [54] with gromos53a6 force field [55]. The simulations were conducted under neutral condition. The modified cyclotide in the complex with gp120 was placed in a dodecahedron box with an edge of 1.0 nm. The box was then solvated with SPC water molecules. The topology file was manually adjusted to create a cyclic cyclotide [35]. Ions were added to neutralize the entire system. Energy minimization was performed on the complex using the 50,000 step of steepest descent algorithm. The equilibration phase was then applied using a 100 ps of NVT ensemble at a temperature of 300 K and a 100 ps of NPT ensemble at 1 bar pressure.

MD simulation of each modified cyclotide was carried out for 20 ns. The temperature was kept constant using the V-rescale temperature coupling algorithm. The pressure coupling was applied at 1 atm under isotropic molecule-based scaling using the Parrinello Rahman method. All bonds were constrained with the LINCS algorithm. The GRID method was used to search and update the neighbors with 10 fs. Particle Mesh Ewald (PME) was used to treat long-range electrostatic energy. Short-range neighbor list cutoff, short-range electrostatic cutoff, and short-range van der Waals (VDW) cutoff were set to 0.9, 0.9, and 1.4, respectively.

### MD simulation of CD4M33

Before the simulation, the structure and topology files of the CD4-mimetic miniprotein CD4M33 were prepared. To build CD4M33 in complex with gp120 (1GC1), the CD4M33 structure was separated from its original complex (PDB ID: 1YYL) and placed at the CD4 binding site of gp120. The topology of unnatural amino acids of CD4M33 (thiopropionic acid and biphenylalanine) were built separately using the Automated Topology Builder (ATB) [56]. The simulation condition was the same as that used in the simulations of cyclotides.

### Analysis of the MD simulation results

The g_dist, g_rmsf, and g_rms Gromacs’s modules were used to determine the distance between the groups, RMSF and RMSD, respectively, for the entire simulation. The interaction energy was extracted from Gromacs energy file using g_energy tool. Interaction energy is the sum of non-bonded interaction energy based on gromos53a6 force field. To decompose the non-bonded interaction energy between the protein and the peptide, the simulation was rerun using a plain cut-off to treat long-range electrostatic energy. The number of hydrogen bonds was determined by g_hbond tool. The analysis was performed over last 5 ns of the simulation.

## Supporting Information

S1 Fig Chart of the distance between the center of mass of gp120 and peptide inhibitors in 20 ns simulation. Y axes represents the distance in nm and X axes represents simulation time in 20 ns
(PDF)

S2 Fig Chart of backbone root-mean-square deviation (BB-RMSD) of gp120 in the complex with peptide inhibitors in 20 ns simulation. BB-RMSD of gp120 is shown in black line and BB-RMSD of peptide molecules i.e. native cyclotide, modified cyclotide and cd4m33 are shown in red line. Y axes represents the distance in nm and X axes represents simulation time in 20 ns
(PDF)

S3 Fig Chart of backbone root-mean-square fluctuation (BB-RMSF) of gp120 in the complex with peptide inhibitors. BB-RMSF of native cyclotide, modified cyclotide and cd4m33 in complex with gp120 are shown in black line. Y axes represents the distance in nm and X axes represents the residue number.
(PDF)

S1 Table The hotspot-containing peptide library derived from CD4 amino acid sequence. The residue that were estimated the binding energy lower than -2.0 kcal/mol are emphasized in red, and the residue that were estimated the binding energy lower than -0.5 kcal/mol but higher than -2.0 kcal/mol are emphasized in blue.
(PDF)

S2 Table Top 10 candidates ranked by Rosetta docking score. The GA modification process was carried out of 100 generations. The top 10 candidates were selected from all 910 modified structures.
(PDF)

S3 Table Non-bonded energy and hydrogen bond contribution of each residue of GA763 in the interaction with gp120 investigated in last 5 ns of 20 ns simulation. The interaction energy was calculated from the sum of average electrostatic and Van Der Waal (VDW) energy in the last 5 ns in a 20 ns MD simulation. The number of H-bond was the average number of H-bond per time frame in the last 5 ns.
(PDF)

S4 Table Non-bonded energy and hydrogen bond contribution of each residue of GA190 in the interaction with gp120 investigated in last 5 ns of 20 ns simulation. The interaction energy was calculated from the sum of average electrostatic and Van Der Waal (VDW) energy in the last 5 ns in a 20 ns MD simulation. The number of H-bond was the average number of H-bond per time frame in the last 5 ns.
(PDF)

S1 Text Pseudo code of chromosome construction and translation. Function Construct_chromosome generates chromosome as a fixed list. Function Translate_chromosome takes the chromosome as input and then returns modified cyclotide amino acid sequence as an output.
(PDF)
