# Supplementary figures and images for: Computational Design of Hypothetical New Peptides Based on a Cyclotide Scaffold as HIV gp120 Inhibitor
Source: PLoS One. 2015 Oct 30;10(10):e0139562. doi: 10.1371/journal.pone.0139562 (PMC4627658; doi:10.1371/journal.pone.0139562)

KB1

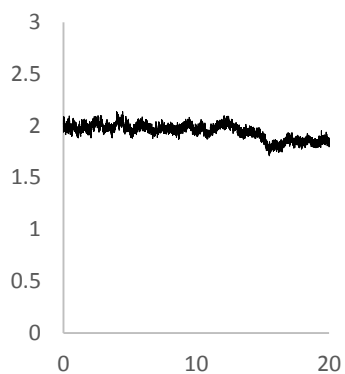

cd4m33

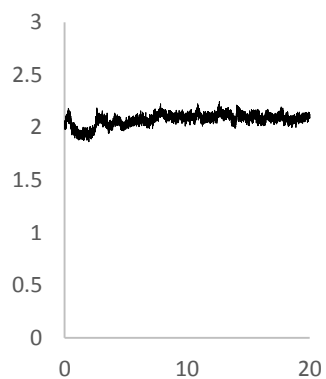

GA61

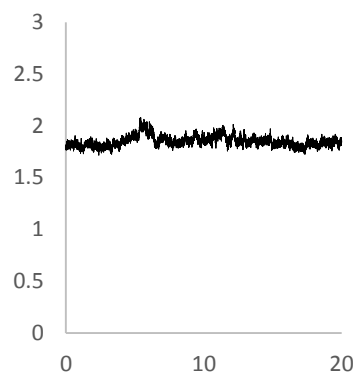

GA763

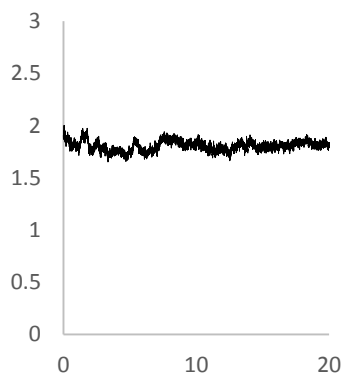

GA218

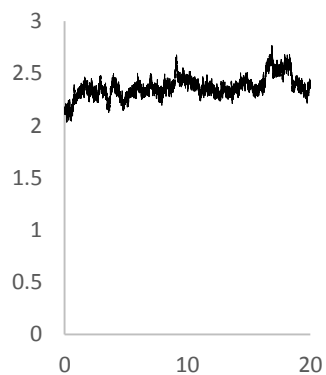

GA190

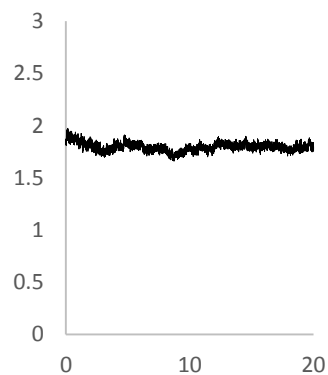

GA689

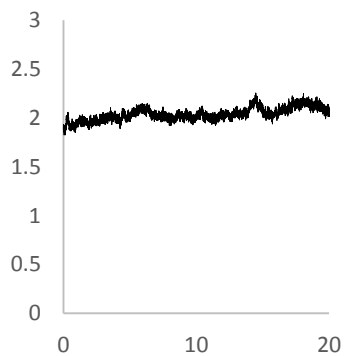

GA479

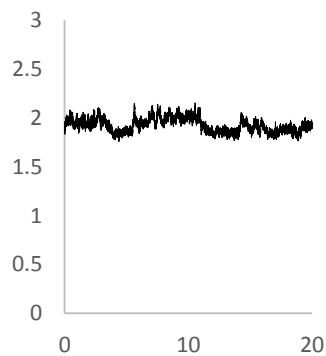

Supplement: S1 Fig — Y axes represents the distance in nm and X axes represents simulation time in 20 ns (PDF) [file pone.0139562.s001.pdf]

KB1

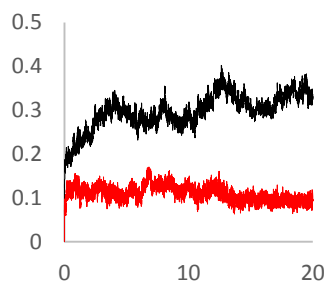

cd4m33

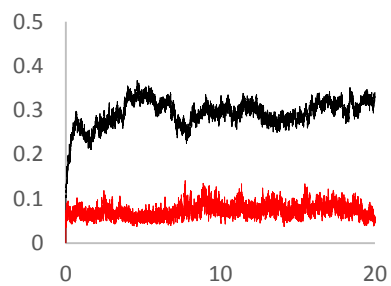

GA61

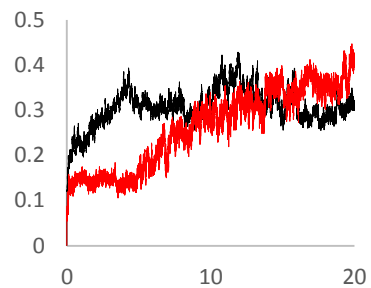

GA763

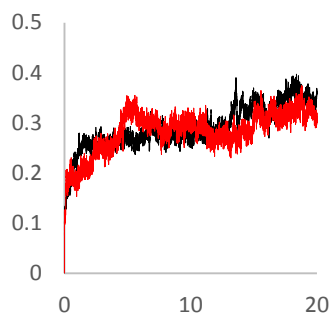

GA218

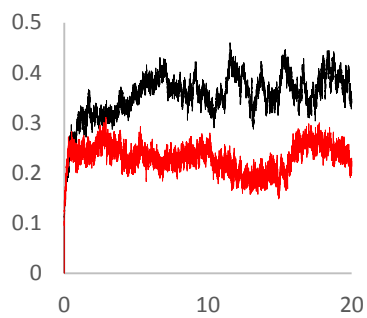

GA190

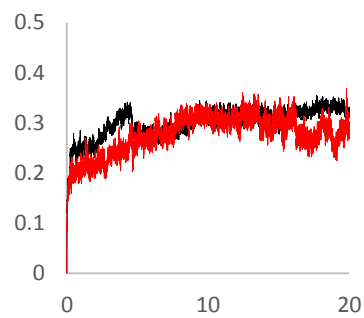

GA689

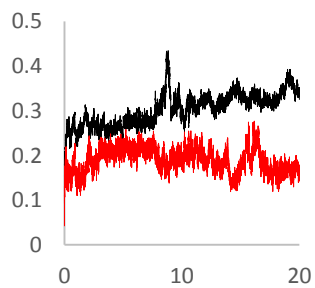

GA479

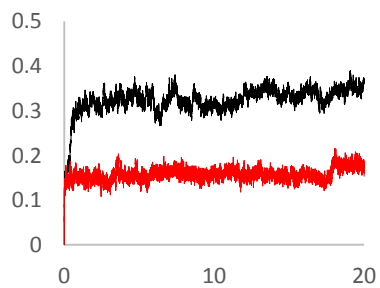

Supplement: S2 Fig — BB-RMSD of gp120 is shown in black line and BB-RMSD of peptide molecules i.e. native cyclotide, modified cyclotide and cd4m33 are shown in red line. Y axes represents the distance in nm and X axes represents simulation time in 20 ns (PDF) [file pone.0139562.s002.pdf]

KB1

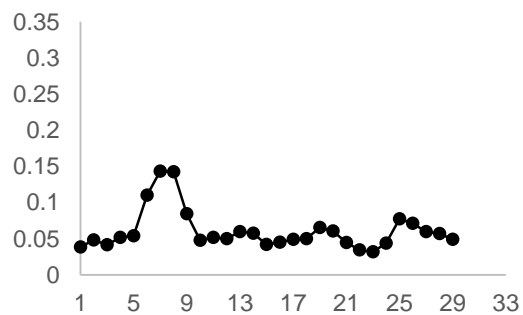

GA61

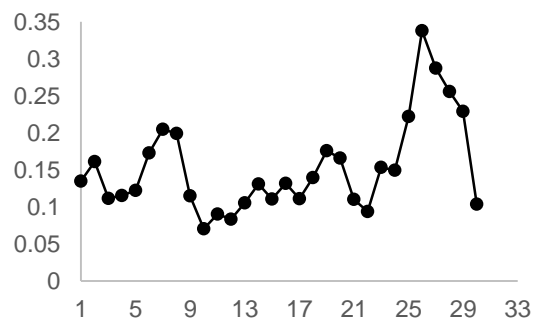

GA763

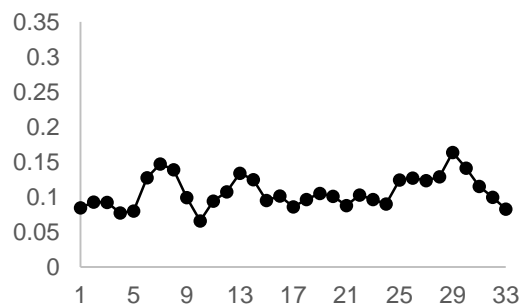

GA218

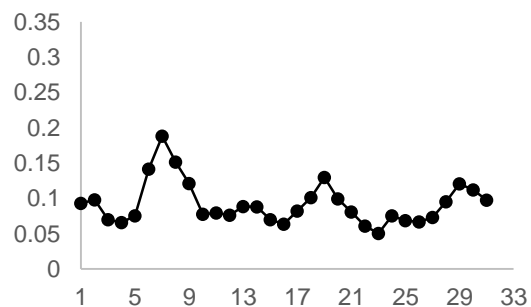

GA190

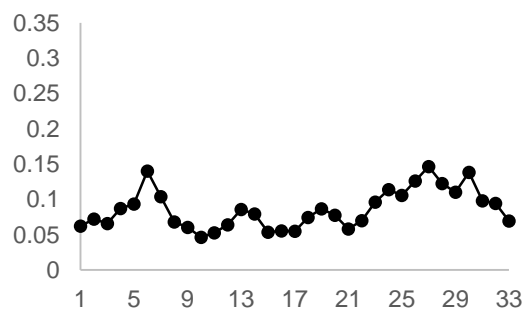

GA689

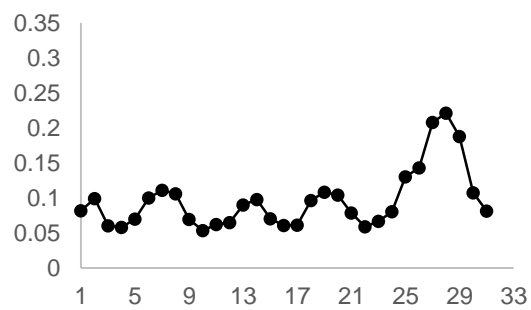

GA479

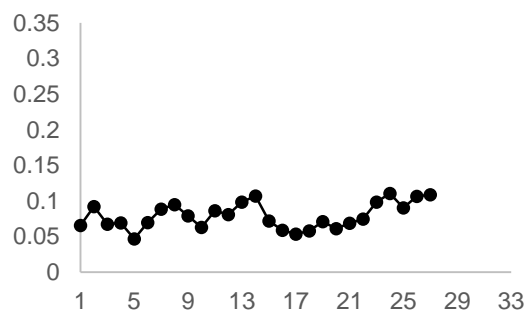

Supplement: S3 Fig — BB-RMSF of native cyclotide, modified cyclotide and cd4m33 in complex with gp120 are shown in black line. Y axes represents the distance in nm and X axes represents the residue number. (PDF) [file pone.0139562.s003.pdf]
