# Supplementary material for: Computational Design of Hypothetical New Peptides Based on a Cyclotide Scaffold as HIV gp120 Inhibitor
Source: PLoS One. 2015 Oct 30;10(10):e0139562. doi: 10.1371/journal.pone.0139562 (PMC4627658; doi:10.1371/journal.pone.0139562)
Supplement: S1 Table — The residue that were estimated the binding energy lower than -2.0 kcal/mol are emphasized in red, and the residue that were estimated the binding energy lower than -0.5 kcal/mol but higher than -2.0 kcal/mol are emphasized in blue. (PDF) [file pone.0139562.s004.pdf]

Sequence	Residue name of CD4
QFL	Gln40- Phe43- Leu44
FLT	Phe43-Leu44-Thr45
FLTKG	Phe43-Leu44-Thr45-Lys46-Gly47
FLTKGPSK	Phe43-Leu44-Thr45-Lys46-Gly47-Pro48-Ser49-Lys50
GSF	Gly41-Ser42-Phe43
QGSF	Gln40-Gly41-Ser42-Phe43
QGSFL	Gln40-Gly41-Ser42-Phe43-Leu44
RS	Arg59-Ser60
FRS	Phe43-Arg59-Ser60
RSLW	Arg59-Ser60-Leu61-Trp62
RSLWDQ	Arg59-Ser60-Leu61-Trp62-Asp63-Gln64
NQI	Asn32-Gln33-Ile34
KNSNQI	Lys29-Asn30-Ser31-Asn32-Gln33-Ile34
RF	Arg59-Phe43
RFLT	Arg59-Phe43-Leu44-Thr45
RFLTKG	Arg59-Phe43-Leu44-Thr45-Lys46-Gly47
F	Phe43
SF	Ser42-Phe43
SFL	Ser42-Phe43-Leu44
GSFLT	Gly41-Ser42-Phe43-Leu44-Thr45
