# Supplementary material for: Computational Design of Hypothetical New Peptides Based on a Cyclotide Scaffold as HIV gp120 Inhibitor
Source: PLoS One. 2015 Oct 30;10(10):e0139562. doi: 10.1371/journal.pone.0139562 (PMC4627658; doi:10.1371/journal.pone.0139562)
Supplement: S2 Table — The GA modification process was carried out of 100 generations. The top 10 candidates were selected from all 910 modified structures. (PDF) [file pone.0139562.s005.pdf]

Amino acid sequence	Name	Docking score (Rosetta unit)
CGETCVGGTCNTPGCTCSWRSLWVSFLCLP	GA767	-7.95
CGETCVGGTCNTPGCTCSPVCRFRQGSFGL	GA61	-7.90
CGETCVGGTCNTPGCTCSWPVCTRFNQGSFGLP	GA52	-7.62
CGETCVGGTCNTPGCTCWPVCGSFLRFLTKGPV	GA763	-7.59
CGETCVGGTCNTPGCTCRFSWPCTQGSFGLP	GA218	-7.44
CGETCVGGTCNTPGCTCWPVCGSFLTGQGSFPV	GA190	-7.33
CGETCVGGTCNTPGCTCSWPVCNRFGSFLTP	GA689	-7.13
CGETCVGGTCNTPGCTCSFLTKGVCSFLN	GA270	-7.08
CGETCVGGTCNTPGCTCSQGSFWPVCRFTNGLPV	GA32	-7.01
CGETCVGGTCNTPGCTCWPCSFGSFNV	GA479	-6.98
