# Supplementary material for: Computational Design of Hypothetical New Peptides Based on a Cyclotide Scaffold as HIV gp120 Inhibitor
Source: PLoS One. 2015 Oct 30;10(10):e0139562. doi: 10.1371/journal.pone.0139562 (PMC4627658; doi:10.1371/journal.pone.0139562)
Supplement: S3 Table — The interaction energy was calculated from the sum of average electrostatic and Van Der Waal (VDW) energy in the last 5 ns in a 20 ns MD simulation. The number of H-bond was the average number of H-bond per time frame in the last 5 ns. (PDF) [file pone.0139562.s006.pdf]

Residue number	Residue abbreviation	Non-bonded interaction (kJ/mol)			Interaction contribution (%)	Ave H-bond
		Electrostatic energy	VDW energy	Total		
1	C	4.5	-8.5	-4.0	0.4	0.0
2	G	1.7	-3.5	-1.8	0.2	0.0
3	E	-10.2	-11.3	-21.6	2.0	0.0
4	T	0.3	-1.6	-1.3	0.1	0.0
5	C	-0.8	-1.5	-2.3	0.2	0.0
6	V	7.0	-5.7	1.3	-0.1	0.0
7	G	-4.5	-5.1	-9.6	0.9	0.0
8	G	-20.5	-9.8	-30.3	2.8	0.2
9	T	-32.0	-17.0	-49.0	4.6	0.4
10	C	2.3	-3.9	-1.6	0.1	0.0
11	N	-24.2	-6.7	-31.0	2.9	0.0
12	T	-0.7	-18.4	-19.1	1.8	0.0
13	P	0.6	-34.6	-34.0	3.2	0.0
14	G	-16.5	-16.7	-33.2	3.1	0.2
15	C	-10.3	-14.4	-24.7	2.3	0.0
16	T	-34.4	-25.7	-60.1	5.6	0.4
17	C	6.5	-8.8	-2.2	0.2	0.0
18	W	-77.1	-15.1	-92.2	8.6	1.3
19	P	0.2	-1.2	-1.1	0.1	0.0
20	V	-4.4	-0.9	-5.3	0.5	0.0
21	C	-1.6	-1.5	-3.1	0.3	0.0
22	G	3.6	-1.2	2.5	-0.2	0.0
23	S	1.1	-1.6	-0.5	0.1	0.0
24	F	-0.8	-12.8	-13.7	1.3	0.0
25	L	0.4	-2.7	-2.3	0.2	0.0
26	R	-24.8	-1.5	-26.2	2.5	0.0
27	F	-0.8	-18.6	-19.4	1.8	0.0
28	L	-1.5	-5.8	-7.3	0.7	0.0
29	T	-10.8	-8.4	-19.2	1.8	0.0
30	K	-431.8	-23.8	-455.7	42.7	0.6
31	G	-5.6	-17.4	-23.0	2.2	0.5
32	P	-20.9	-32.5	-53.4	5.0	0.0
33	V	-11.4	-11.7	-23.1	2.2	0.0
Summations		-717.7	-349.7	-1067.4	100.0	3.7
