# Supplementary material for: Computational Design of Hypothetical New Peptides Based on a Cyclotide Scaffold as HIV gp120 Inhibitor
Source: PLoS One. 2015 Oct 30;10(10):e0139562. doi: 10.1371/journal.pone.0139562 (PMC4627658; doi:10.1371/journal.pone.0139562)
Supplement: S4 Table — The interaction energy was calculated from the sum of average electrostatic and Van Der Waal (VDW) energy in the last 5 ns in a 20 ns MD simulation. The number of H-bond was the average number of H-bond per time frame in the last 5 ns. (PDF) [file pone.0139562.s007.pdf]

Residue number	Residue abbreviation	Non-bonded interaction (kJ/mol)			Interaction contribution (%)	Ave H-bond
		Electrostatic energy	VDW energy	Total		
1	C	-3.5	-8.5	-12.0	1.3	0.0
2	G	-15.0	-4.0	-19.1	2.0	0.6
3	E	-3.1	-5.2	-8.3	0.9	0.0
4	T	0.1	-0.3	-0.2	0.0	0.0
5	C	-0.1	-1.0	-1.1	0.1	0.0
6	V	7.1	-1.0	6.1	-0.6	0.0
7	G	-4.6	-6.3	-10.8	1.1	0.0
8	G	-30.0	-10.6	-40.7	4.2	0.0
9	T	-95.5	-4.7	-100.2	10.4	1.9
10	C	-1.2	-2.0	-3.2	0.3	0.0
11	N	-0.2	-2.3	-2.5	0.3	0.0
12	T	-13.0	-12.8	-25.8	2.7	0.5
13	P	-13.8	-19.6	-33.4	3.5	0.9
14	G	5.6	-14.1	-8.5	0.9	0.1
15	C	7.5	-6.6	0.9	-0.1	0.0
16	T	-11.7	-14.7	-26.3	2.7	0.0
17	C	-0.7	-8.6	-9.3	1.0	0.0
18	W	-17.9	-48.4	-66.3	6.9	0.6
19	P	-1.1	-8.9	-10.0	1.0	0.0
20	V	0.5	-1.6	-1.1	0.1	0.0
21	C	0.7	-1.3	-0.6	0.1	0.0
22	G	-4.4	-1.8	-6.2	0.6	0.0
23	S	-50.3	-5.4	-55.8	5.8	0.8
24	F	-31.8	-49.6	-81.5	8.5	0.4
25	L	12.2	-27.9	-15.6	1.6	0.0
26	T	-41.7	-16.3	-58.1	6.1	0.4
27	G	-47.0	-19.7	-66.8	7.0	0.2
28	Q	-149.9	-19.3	-169.2	17.6	1.9
29	G	-56.6	-1.5	-58.1	6.1	1.0
30	S	-12.9	-4.3	-17.1	1.8	0.1
31	F	-2.0	-13.7	-15.6	1.6	0.0
32	P	-7.9	-11.9	-19.8	2.1	0.5
33	V	-2.1	-21.0	-23.1	2.4	0.0
Summations		-584.2	-374.9	-959.1	100.0	9.9
