# Supplementary material for: Computational Design of Hypothetical New Peptides Based on a Cyclotide Scaffold as HIV gp120 Inhibitor
Source: PLoS One. 2015 Oct 30;10(10):e0139562. doi: 10.1371/journal.pone.0139562 (PMC4627658; doi:10.1371/journal.pone.0139562)
Supplement: S1 Text — Function Construct_chromosome generates chromosome as a fixed list. Function Translate_chromosome takes the chromosome as input and then returns modified cyclotide amino acid sequence as an output. (PDF) [file pone.0139562.s008.pdf]

```
FUNCTION Construct_chromosome
    FOR gene_index = 0 to 10
        chromosome[gene_index]= random in range(0 to 1)
    END FOR
    chromosome[11]= random in range(0 to 12)
    chromosome[12]= random in range(0 to 20)
    chromosome[13]= random in range(0 to 13)
    chromosome[14]= random in range(0 to 20)
    RETURN chromosome
END FUNCTION


FUNCTION Translate_chromosome
    cyclotide_sequence= 'CGETCVGGTCNTPGCTCSWPVCTRNGLPV'
    OPEN Epitope_library_file
    epitope[ ] = READ Epitope_library_file
    chromosome = CALL FUNCTION Construct_chromosome
    FOR gene_index = 0 to 3
        IF chromosome[gene_index] equal to zero THEN
            cyclotide_sequence[17+gene_index] =  ' – '
            //Loop5 starts at residue 17
        END IF
    END FOR
    FOR gene_index = 4 to 10
        count=0
        IF chromosome[gene_index] equal to zero THEN
            cyclotide_sequence[22+count] =  ' – '
            //Loop6 starts at residue 22
        END IF
        count=count+1
    END FOR
    INSERT epitope[gene_index[12]] to cyclotide_sequence[gene_index[11]+17]
    INSERT epitope[gene_index[14]] to cyclotide_sequence[gene_index[13]+17]
    DELETE ' - ' in cyclotide_sequence
    RETURN cyclotide_sequence
END FUNCTION
```
